# Differences in the Activity of Endogenous Bone Morphogenetic Protein Signaling Impact on the Ability of Induced Pluripotent Stem Cells to Differentiate to Corneal Epithelial‐Like Cells

**DOI:** 10.1002/stem.2750

**Published:** 2017-12-21

**Authors:** Taty Anna Kamarudin, Sanja Bojic, Joseph Collin, Min Yu, Sameer Alharthi, Harley Buck, Alex Shortt, Lyle Armstrong, Francisco C. Figueiredo, Majlinda Lako

**Affiliations:** ^1^ Institute of Genetic Medicine, International Centre for Life, Newcastle University, Central Parkway Newcastle upon Tyne United Kingdom; ^2^ Princess Al Jawhara Al‐Brahim Center of Excellence in Research of Hereditary Disorders, King Abdulaziz University Saudi Arabia; ^3^ UCL Institute of Immunology and Transplantation, Royal Free Campus London United Kingdom; ^4^ Department of Ophthalmology Royal Victoria Infirmary, Queen Victoria Road Newcastle upon Tyne United Kingdom

**Keywords:** Human embryonic stem cell, Human induced pluripotent stem cell, Corneal epithelial progenitors, Corneal epithelial cells, Bone morphogenetic protein 4, Retinoic acid, Epidermal growth factor

## Abstract

Cornea is a clear outermost layer of the eye which enables transmission of light onto the retina. The transparent corneal epithelium is regenerated by limbal stem cells (LSCs), whose loss/dysfunction results in LSCs deficiency (LSCD). Ex vivo expansion of autologous LSCs obtained from patient's healthy eye followed by transplantation onto the LSCs damaged/deficient eye, has provided a successful treatment for unilateral LSCD. However, this is not applicable to patient with total bilateral LSCD, where LSCs are lost/damaged from both eyes. We investigated the potential of human induced pluripotent stem cell (hiPSC) to differentiate into corneal epithelial‐like cells as a source of autologous stem cell treatment for patients with total bilateral LSCD. Our study showed that combined addition of bone morphogenetic protein 4 (BMP4), all trans‐retinoic acid and epidermal growth factor for the first 9 days of differentiation followed by cell‐replating on collagen‐IV‐coated surfaces with a corneal‐specific‐epithelial cell media for an additional 11 days, resulted in step wise differentiation of human embryonic stem cells (hESC) to corneal epithelial progenitors and mature corneal epithelial‐like cells. We observed differences in the ability of hiPSC lines to undergo differentiation to corneal epithelial‐like cells which were dependent on the level of endogenous BMP signaling and could be restored via the activation of this signaling pathway by a specific transforming growth factor β inhibitor (SB431542). Together our data reveal a differential ability of hiPSC lines to generate corneal epithelial cells which is underlined by the activity of endogenous BMP signaling pathway. Stem Cells *2018;36:337–348*

**Significance Statement**

Directing differentiation of human induced pluripotent stem cells (hiPSC) to corneal epithelial progenitors could provide an autologous source for cell replacement therapy in patients with bilateral limbal stem cell deficiency, avoiding dependency on post‐transplant immunosuppressant or donated cornea. This work describes a simple and efficient two‐step method with the supplementation of growth factors and small molecules that results in differentiation of pluripotent stem cells to corneal epithelial cells in 20 days. There were cell line differences in capacity to differentiate to corneal epithelial progenitors which were underlined by the endogenous BMP signaling activity and could be restored using SB431542.

## Introduction

Cornea is the transparent region at the front of the eye which enables transmission of light to the retina. It comprises the corneal epithelium, stroma, and endothelium. The corneal epithelium is continuously regenerated by limbal stem cells (LSCs) 1, 2, which migrate from peripheral to central region of the cornea and ascend from basal to superficial layer to differentiate and form a new stratified layer of non‐keratinized squamous epithelium 3. The corneal epithelium develops from surface ectoderm 4, while the stroma and endothelium developed from the mesenchymal tissue and neural crest cells 5.

LSCs deficiency (LSCD) is a disease caused by the loss or dysfunction of LSCs, leading to loss of corneal epithelial integrity and function, often resulting in persistent pain and severe visual impairment 6. Work done by our group and others have shown that the transplantation of ex vivo expanded autologous LSCs is able to reconstruct the corneal surface and to restore vision in patients with unilateral total LSCD 7, 8, 9. However, this treatment is not applicable to a significant number of patients with total bilateral LSCD where patient's both eyes are devoid of LSCs which are needed for the ex vivo expansion and subsequently used for transplantation. Hence, alternative sources of cells that could be used to replace the missing LSCs in total bilateral LSCD are being sought after by many researchers. Of those, transplantation of ex vivo expanded autologous oral mucosa epithelial (OME) cells has been the most used cell source in clinical studies of bilateral LSCD treatment with a reported “success” rate of 48%–75% within follow‐up times up to 34 months 10, 11, 12, 13, 14, 15, 16, 17. Our group provided proof of concept by transplanting autologous ex vivo expanded OME in two patients with histologically confirmed total bilateral LSCD which resulted in successful reversal of LSCD in the treated eye up to 24 months 18. Notwithstanding, we also showed that cultured oral epithelial cells retained a gene expression profile that was attributed to epithelial stem cells in general, but they did not acquire a typical limbal expression pattern after 10–14 days in culture 18, thus indicating that the transplanted cells did not fully transdifferentiate into corneal epithelium.

Recent advances in somatic cell induced reprogramming have shown that it is possible to reprogram somatic cells back to an “embryonic‐like cells” through overexpression of four key pluripotency factors. These are named induced pluripotent stem cells and like human embryonic stem cells (hESC), they are characterized by unlimited self‐renewal and potential to differentiate into any cell type of the adult organism 19, 20. The most important advantage of human induced pluripotent stem cells (hiPSC) is the ability to avoid post‐transplantation rejection by patient's own immune system 21. Traditionally, differentiation of hESC and hiPSC to corneal epithelial cells has relied on usage of feeder cells, undefined conditioned media, or amniotic membrane 22, 23, 24, 25, 26. More recently, small molecule driven protocols have become available resulting in generation of corneal epithelial‐like cells within 6 weeks 27. Bioengineered medical grade collagen matrices have also been shown to provide an excellent carrier for pluripotent stem cell derived limbal epithelial cells, which retained their ability to proliferate when in contact with matrix as well as the ability to differentiate into epithelial cells 28. In this article, we describe the development of a defined feeder‐free monolayer differentiation method which results in differentiation of hESC and hiPSC to corneal epithelial progenitors and mature corneal epithelial cells within 20 days. Furthermore, we show differences in the ability of hiPSC lines to generate corneal epithelial‐like cells which are dictated by the activity of endogenous bone morphogenetic protein (BMP) signaling pathway.

## Materials and Methods

### Cell Culture

Undifferentiated hESC (H9) and hiPSC that were generated and fully characterized in our group (SB‐Ad2 and SB‐Ad3 29) were maintained in BD Matrigel (growth factor reduced) (BD Biosciences, Belgium) with mTeSR1 (STEMCELL Technologies, Cambridge, MA) at 37°C and 5% CO_2_. hESC and hiPSC were passaged every 3–4 days by using 0.02% EDTA (Versene, Belgium) at 1:3–1:6 ratio. 3T3 fibroblasts for feeder plate preparation were maintained in cell culture flasks in fibroblast medium at 37°C and 5% CO_2_. 3T3 fibroblast medium was prepared by mixing 89% high glucose Dulbecco's modified Eagle medium + GlutaMAX; 10% fetal bovine serum (FBS); 1% Pen/Strep (Gibco, UK). 3T3 fibroblasts were passaged every 3 to 4 days using 0.05% Trypsin‐EDTA (Gibco, UK). All cells used in this study were between passages 15 and 50 and assessed to be karyotypically normal through CytoSNP analysis.

### Differentiation of hESC and hiPSC to Corneal Epithelial Cells

**Figure 1:**
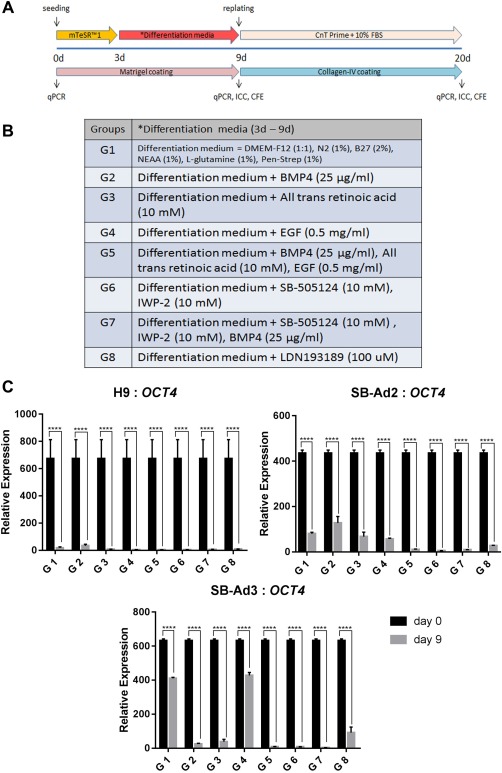
Schematic outline of the differentiation process. The differentiation process is divided into two stages, early (days 0–9) and advanced (days 10–20) **(A)**. Endpoint analyses included quantitative real‐time polymerase chain reaction (qRT‐PCR), immunocytochemistry, and colony forming efficiency. List of differentiation induction media components for each of the eight groups **(B)**. Downregulation of *OCT4* from days 0 to 9 for all the groups in the three cell lines used (H9, SB‐Ad2, and SB‐Ad3) assessed by qRT‐PCR **(C)**. Data are presented as mean ± SEM, *n* = 3. *, statistically different compared with day 0. ****, *p* < .0001. Abbreviations: BMP4, bone morphogenetic protein 4; CFE, colony forming efficiency; EGF, epidermal growth factor; ICC, immunocytochemistry; IWP‐2, Wnt antagonist II; qPCR, quantitative polymerase chain reaction.

A schematic representation of differentiation protocol is presented in Figure 1A. In brief, hESC and hiPSC were seeded at 1.7 × 10^4^ cells per square centimeter 30 on BD Matrigel‐coated plates and kept in mTeSR1 medium for two days. Rho kinase inhibitor, Y27632 (Chemdea, NJ, USA) [10 µM) was added to the mTeSR1 medium for the first 24 hours. Eight differentiation induction media listed in Figure 1B were introduced to the cells according to the respective groups on day 3 and maintained until day 9. The cells were then replated at 1.7 × 10^4^ cells per square centimeter onto 0.05 mg/ml collagen‐IV–coated plates 22 on day 9 and supplemented with CnT‐Prime medium (CELLnTEC, Switzerland) and 10% serum for the next 11 days. Three technical and three biological repeats were set up for each differentiation group.

### Optimization of hiPSC Differentiation Using SB431542

hiPSC were seeded at 1.7 × 10^4^ cells per square centimeter on BD Matrigel coated plates and kept in mTeSR1 medium for 2 days, with 10 µM Rho kinase inhibitor, Y27632 (Chemdea, NJ, USA) added for the first 24 hours. The SB431542 (10 µM) (Tocris, UK) was added to the differentiation media from day 3 for 1, 2, or 3 days. The cell were replated at 1.7 × 10^4^ cells per square centimeter onto collagen‐IV‐coated plates at day 9 and supplemented with CnT‐Prime medium (CELLnTEC, Switzerland) and 10% serum for the next 11 days. Three technical and three biological repeats were set up for each differentiation group.

### RNA Extraction, Reverse Transcription, and Quantitative Real‐Time Polymerase Chain Reaction

RNA was extracted from the cells collected from differentiating hESC and hiPSC at days 0, 9, and 20 using the ReliaPrep RNA Cell Miniprep System (Promega, WI). The RNA quality was evaluated using the Nanodrop 2000 spectrophotometer (Thermo Fisher Scientific, MA). One microgram of extracted RNA was converted into cDNA using reverse transcription (GoScript Transcription System; Promega, WI) following the manufacturer's instructions. Quantitative real‐time polymerase chain reaction (qRT‐PCR) was carried out using the QuantStudio 7 Flex Real‐Time PCR System (Thermo Fisher Scientific, MA) and GoTaq qPCR Master Mix (Promega, WI) according to manufacturer's instructions. The primer sequences used are listed in Supporting Information Table S1. The data were analyzed using the ΔΔCt calculation method.

### Colony Forming Efficiency Assay

3T3 cells were mitotically inactivated with mitomycin C (10 µg/ml) (Sigma‐Aldrich, Germany) for 2 hours as described by Ahmad et al. 22. Then 2.4 × 10^4^ per square centimeter of the cells were added into each well of the six‐well gelatin‐coated plates containing fibroblast medium. The plated cells were incubated at 37°C in 5% CO_2_. The feeder plates were used on the following day. On day 9 or day 20, 1,000 cells from each experimental group were added to each feeder well containing limbal epithelial medium 7. The limbal epithelial medium was replaced after 3 days and every other day thereafter. The colony forming efficiency (CFE) plates were kept for 14 days. The CFE plates were fixed in 3.7% formaldehyde for 10 minutes at room temperature. The wells were washed with phosphate‐buffered saline (PBS) once before enough volume of 1% Rhodamine B (Sigma‐Aldrich, Germany) in methanol was added to each well and incubated for 10 minutes. Cell colonies were washed three times with PBS before being observed and counted with the aid of a dissection microscope.

### Immunofluorescence Staining

Cells on day 9 or day 20 of differentiation were dissociated using Tryple express (Gibco, UK) and kept in PBS supplemented with 2% FBS on ice. Cells were cytospun into poly‐l‐lysine‐coated slides, fixed with 4% paraformaldehyde (Sigma‐Aldrich, Germany) for 15 minutes. Nonspecific staining was inhibited by blocking in 5% normal goat serum for 1 hour at room temperature. Primary antibody was added overnight at 4°C followed by washes and incubation with a secondary antibody at room temperature in a dark humidified chamber for 1 hour. Slides were protected from light as much as possible after the addition of secondary antibody to avoid bleaching of the fluorescence signal. Following staining, cells were washed three times with PBS, with each wash lasting for 3 minutes. Vectashield mounting medium (Vector Laboratories, Burlingame, CA) with Hoechst (Thermo Fisher Scientific, UK) (1:2,000) was added carefully before mounting the coverslips. A list of antibodies used and all dilutions are presented in Supporting Information Table S2.

### Microscopy

Cell morphology was observed and digital images were taken using a microscope (Zeiss AxioVert 1, Germany). ImageJ software was then used to count the stained and unstained cells from the pictures. Five fields, each containing more than 100 cells, were counted for each group.

### Transfection of BMP Reporter Plasmid

Lipofectamine 3000 reagent (Thermo Fisher, Waltham, MA) was used for BMP reporter plasmid (Addgene, MA) transfection. Cells were disassociated by incubating with EDTA (0.02%) for 5 minutes. The disassociated cells were collected and centrifuged at 500*g* for 5 minutes. The cell pellet was resuspended into 2 ml of media and cell count was performed before replating cells at the density of 1.3 × 10^5^ cells into one well of a BD Matrigel coated 24 well plate one day before lipofection. For plasmid lipofection, 500 ng of pGL3‐Basic or pGL3 BRE Luciferase (Promega, Madison, WI) were used to transfect the cells in each well of 24‐well plate following manufacturer's recommendations. Cells that were transfected with empty vector (pGL3‐Basic) or BMP reporter (pGL3 BRE Luciferase) were cotransfected with empty Renilla vector (pRL‐Null) (Promega, Madison, WI).

### Luciferase Assays

Transfected cells were cultured in mTeSR1 alone or mTeSR1 supplemented with BMP4 or BMP4 and SB431542. Cell extracts were prepared 48 hours after transfection using a passive lysis buffer. Luciferase activities were evaluated with a Dual‐Luciferase Assay System (Promega, Madison, WI) according to the manufacturer's recommendations using Varioskan LUX plate reader (Thermo Fisher Scientific, Waltham, MA). Background luminescence was determined using untransfected cells and the background readings were then subtracted from the resulting luminescence readings before being normalized to Renilla luminescence and presented as relative luminescence unit.

### Statistical Analysis

Statistical analysis was performed with one‐way analysis of variance analysis with GraphPad Prism 7 software. Unless otherwise stated in all figures data are shown as mean ± SEM (*n* = 3). *, *p* < .05; **, *p* < .01; ***, *p* < .001; ****, *p* < .0001.

## Results

### Differentiation of hESC and hiPSC to Corneal Epithelial Progenitors

One hESC (H9) and two hiPSC (SB‐Ad2 and SB‐Ad3) lines were directed to differentiate to corneal epithelial‐like cells using a two stage differentiation protocol which aimed at generating corneal epithelial progenitors (days 0–9) and their further differentiation to mature corneal epithelial cells (days 10–20). In the first 9 days, the serum‐free medium was supplemented with a range of growth factors and signaling molecules which included BMP4 and all‐trans retinoic acid (RA) known to promote non‐neural ectodermal differentiation 31, 32, and epidermal growth factor (EGF) which stimulates the proliferation of corneal epithelial progenitors 33. Inhibition of Wnt and transforming growth factor (TGF) β pathway have been reported to be necessary for guiding differentiation of hESC and hiPSC to a ventral neural fate and eye field determination; hence, we included IWP‐2, a Wnt/β‐catenin pathway inhibitor, and SB505124, a selective TGFβ type 1 receptor inhibitor, either on their own or in combination with BMP4 in groups 6 and 7, respectively 27. We also included one additional group with a BMP pathway inhibitor (LDN 193189) to control for the impacts of BMP4, which we used to induce commitment to non‐neural ectoderm. A total of seven combinations of growth factors and signaling molecules were tested, along with the basal medium alone in three independent differentiation experiments for each cell line. The differentiation induction media and the plate coating were changed on day 9 to CnT‐Prime with 10% fetal bovine serum and collagen‐IV coating, respectively, to promote the differentiation of corneal epithelial progenitors into mature corneal epithelial‐like cells. A Schematic outline of the differentiation protocol is detailed in Figure 1A and 1B.

We noticed similar morphological changes observed for both hESCs and hiPSC (Supporting Information Fig. S1A–S1C). The experimental groups 2, 3, and 5 showed the most differentiated morphology typical of epithelial cells on day 9 for both hESC and hiPSC (Supporting Information Fig. S1A–S1C, black arrows). The differentiated cells grew into pockets of flatter and larger cells with higher cytoplasm to nucleus ratio in between the smaller undifferentiated stem cells, corroborating similar observations published by Xu et al. 34. In contrast, most cells in the experimental groups 1, 4, 6, and 8 proliferated robustly and retained the small and compact cell appearance. qRT‐PCR analysis indicated that on day 9, all experimental groups across hESC and hiPSC differentiations were associated with loss of the pluripotent phenotype as shown by a significant decrease in the expression of *OCT4* (Fig. 1C).

**Figure 2:**
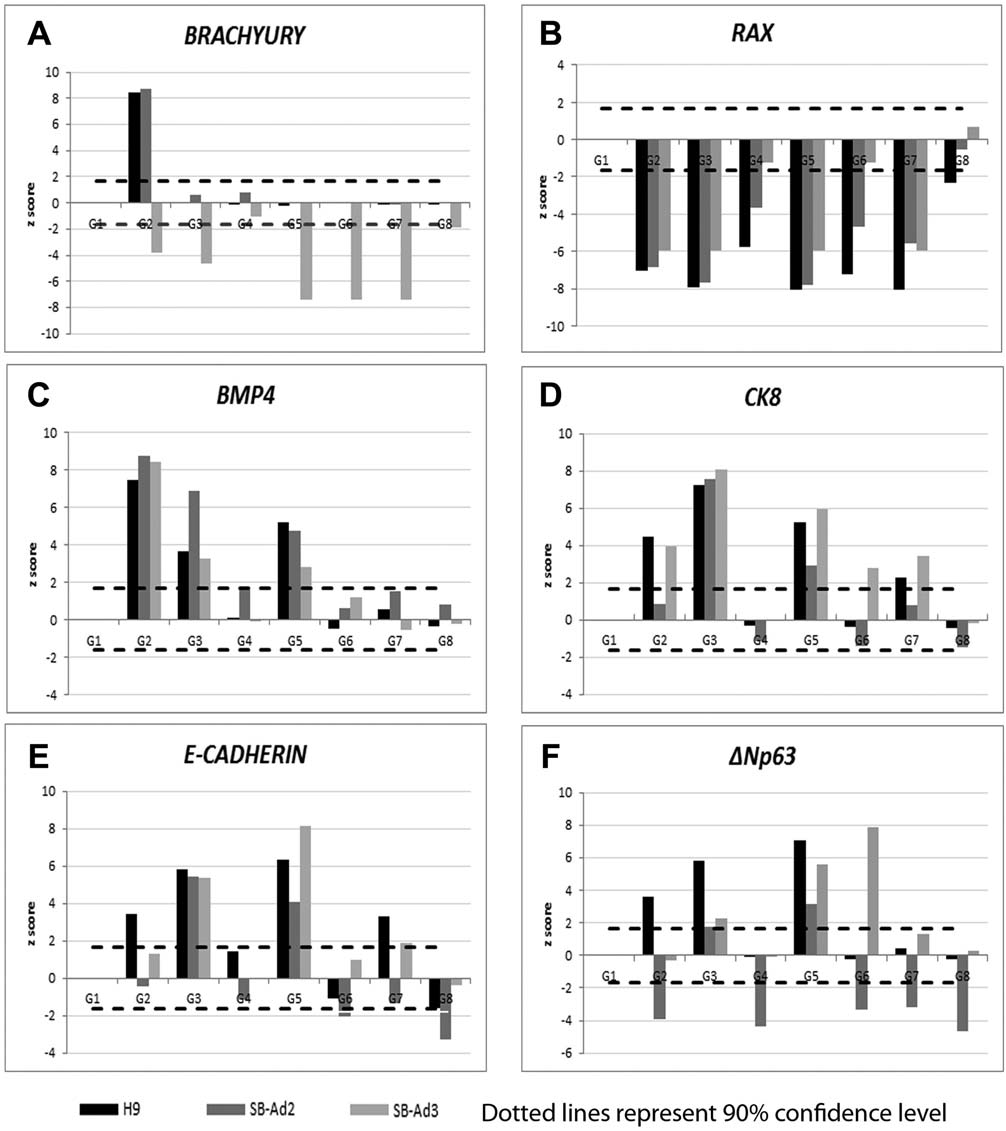
Bone morphogenetic protein 4 (BMP4), retinoic acid, and epidermal growth factor improve early corneal/limbal differentiation process. Quantitative real‐time polymerase chain reaction analysis of *BRACHYURY*, *RAX*, *BMP4*, *CK8*, *ECADHERIN*, and *ΔNp63* genes for groups G2–G8 compared with control group (G1) presented as z scores **(A–F)**. z score was calculated using the following formula: z score = D/SEM where D is the difference between the two means and SEM is the standard error of mean (computed from the data). Dotted lines represent 90% confidence level. Abbreviation: BMP4, bone morphogenetic protein 4.

To assess the differentiation efficiency and compare the effects of media supplementation across the eight groups, qRT‐PCR analysis was carried out at day 9 of differentiation. The results for each group were compared with the control group (G1) which contained no growth factors or small molecules supplementation and presented as z scores. Addition of BMP4 has been associated with differentiation of hESC and hiPSC to mesodermal lineages 35; however, a significant increase in the expression of mesodermal marker, *BRACHYURY* was only observed in the hESC (H9) and one hiPSC line (SB‐Ad2; Fig. 2A) upon BMP4 treatment (Group 2). The expression of *RAX*, a gene expressed in the eye primordia and required for retinal cell fate determination 36, was significantly downregulated in groups 2–7 for both hESC and hiPSC, thus indicating that in all these groups, the differentiation to neuroectodermal lineages was avoided (Fig. 2B). *BMP4* is expressed in early ectodermal tissue 32, 37 and is often used as marker of non‐neural ectoderm, developing cornea and lens. Our qRT‐PCR analysis indicated a significant upregulation of *BMP4* in experimental groups 2, 3, and 5 of hESC and two hiPSC (Fig. 2C), suggesting that the differentiation factors added to these three groups encouraged differentiation to non‐neural ectoderm 30. The expression of ectodermal cytokeratin 8 (*CK8*), basal, and suprabasal corneal epithelium (*E‐CADHERIN*) and putative LSCs (*ΔNp63*) markers were all significantly increased in experimental groups 3 and 5 of both hESCs and hiPSC (Fig. 2D–2F), indicating a likely commitment of these groups to corneal epithelial progenitors.

**Figure 3:**
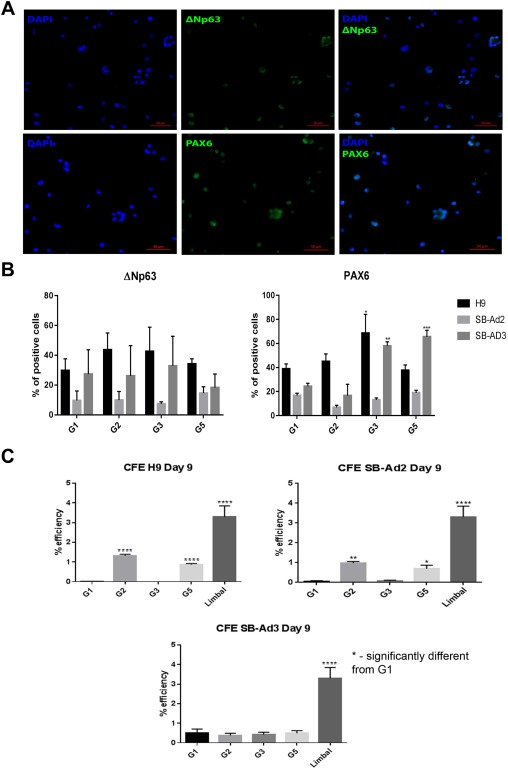
ΔNp63 protein expression and colony forming efficiency. Immunocytochemistry analyses for differentiated cells on day 9 for G1, G2, G3, and G5. Representative immunofluorescence images showing positive ΔNp63 and PAX6 nuclear staining in G3 of H9 cells **(A)** and percentage of ΔNp63 and PAX6 positive cells for H9, SB‐Ad2, and SB‐Ad3 **(B)**. Colony forming efficiency assays for the three cell lines on day 9 for G1, G2, G3 G5, and human limbal epithelium **(C)**. Scale bar = 50 µm. Data are presented as mean ± SEM, *n* = 3. *, statistically different compared with G1. *, *p* < .05; **, *p* < .01; ***, *p* < .001; ****, *p* < .0001. Abbreviations: CFE, colony forming efficiency; DAPI, 4′,6‐diamidino‐2‐phenylindole.

The z scores from the qRT‐PCR analysis consistently indicated that the experimental groups that were supplemented with BMP4, RA, and a combination of BMP4, RA, and EGF showed a significant upregulation of non‐neural ectoderm, epithelial, cell junction, and putative LSC markers. Therefore, we went on to analyze these groups by immunostaining for the expression of putative LSCs protein, ΔNp63. No significant differences between the control nonsupplemented groups and the ones that received BMP4, RA, and a combination of BMP4, RA, and EGF were found (Fig. 3A, 3B). These immunostaining results do not corroborate the qRT‐PCR analysis and a possible reason for this may be the post‐translational modifications already reported for the p63 protein 38. Expression of PAX6, a marker of neuroectodermal 39, anterior placodal ectoderm, and developing eye and lens, was significantly highest in groups treated with RA and a combination of BMP4, RA, and EGF for the differentiating hESC and one of the hiPSC (Fig. 3B).

CFE was highest in experimental groups 2 and 5 of hESC and one of the hiPSC lines (SB‐Ad2, Fig. 3C), suggesting that supplementation of basic media with BMP4 or a combination of BMP4, RA, and EGF provides an optimal combination for directing differentiation of hESC and hiPSC to corneal epithelial progenitor cells. Notwithstanding, no significant difference in CFE ability was observed between the four experimental groups tested in the second hiPSC line (SB‐Ad3), indicating significant differences between the hiPSC lines in their response to our differentiation protocols and the need for further culture modifications for nonresponsive hiPSC lines.

### Differentiation of hESC and hiPSC to Corneal Epithelial‐Like Cells

**Figure 4:**
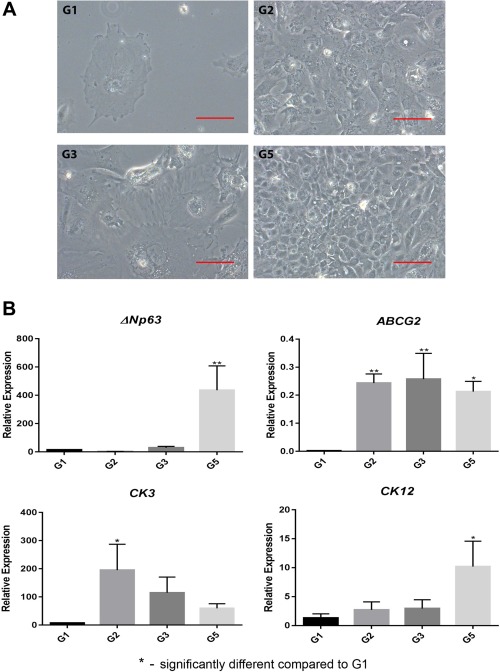
Cell morphology at advanced stage of differentiation and molecular characterization. Differentiated G1, G2, G3, and G5 of H9 cells morphological appearance on day 20 **(A)**, and quantitative real‐time polymerase chain reaction analysis of corneal and limbal epithelial genes **(B)**. Data are presented as mean ± SEM, *n* = 3. Scale bar = 50 µm. *, statistically different compared with G1. *, *p* < .05; **, *p* < .01.

During the second differentiation window (days 10–20), we aimed at promoting the maturation of corneal epithelial progenitors to epithelial cells by replating on collagen‐IV coated plates and feeding the cells with a specific corneal differentiation medium (CnT‐Prime) supplemented with 10% serum (Fig. 1A). Similar morphological changes were observed in both hESC and hiPSC during this time window (Fig. 4A; Supporting Information Figs. 2A, 3A). Cells appeared larger and flatter and characterized by an epithelial‐like morphology by the end of the experiment on day 20. qRT‐PCR analysis showed that the combination of RA, BMP4, and EGF (group 5) was associated with the greatest upregulation of putative LSC marker (*ΔNp63*) across the cell lines (Fig. 4B; Supporting Information Figs. 2B, 3B). Expression of *ABCG2*, which was reported as another putative LSCs marker 40, was also consistently highest in groups supplemented with RA across the cells lines. Differentiated corneal epithelial cytokeratin, *CK3* expression was more variable across the cell lines, with the highest expression observed in BMP4 supplemented group for hESC, RA supplemented group for hiPSC‐SB‐Ad3 and RA and RA, BMP4, and EGF supplemented group for hiPSC‐SB‐Ad2. *CK12* expression was consistently highest in the groups supplemented with RA, BMP4, and EGF. Together, these data suggest some intra‐line differences in the capacity to mature toward corneal epithelial‐like cells.

**Figure 5:**
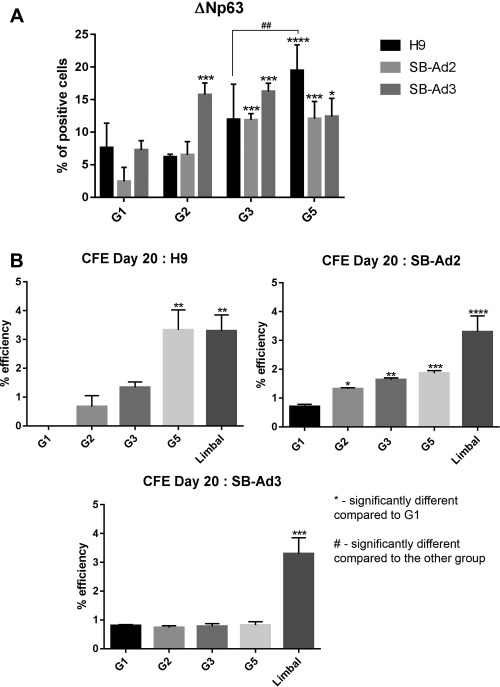
ΔNp63 protein expression and colony forming efficiency at day 20 of differentiation. Percentage of ΔNp63 positive cells for H9, SB‐Ad2, and SB‐Ad3 cells on day 20 for G1, G2, G3, and G5 **(A)**. Colony forming efficiency for the three cell lines on day 20 for G1, G2, G3 G5, and human limbal epithelium **(B)**. Data are presented as mean ± SEM, *n* = 3. *, statistically different compared with G1 of the same cell line. *, *p* < .05; **, *p* < .01; ***, *p* < .001; ****, *p* < .0001. #, statistically different compared with the other group; ##, *p* < .01. Abbreviation: CFE, colony forming efficiency.

Immunostaining analysis at day 20 revealed a significant upregulation of ΔNp63 expression in groups supplemented with BMP4, RA, and EGF across the hESC and hiPSC lines (Fig. 5A). It needs to be noted though that the expression of this marker decreased from day 9 of differentiation, indicating further differentiation of these cells to CK3 and CK12 expressing corneal epithelial cells as shown in Supporting Information Figure 4A and 4B. CFE assays also showed that the BMP4, RA, and EGF supplemented group in hESC resulted in the highest colony forming ability which was similar to human limbal epithelial progenitor cells (Fig. 5B). All the selected groups of one of the hiPSC lines (SB‐Ad2) showed an increased CFE ability compared with control group; however, this was considerably lower than human limbal epithelial progenitor cells (Fig. 5B). In contrast, all the treated groups from the second hiPSC line (SB‐Ad3) showed a very low CFE ability and no difference to the untreated control group, indicating a lack of response from this cell line to differentiation factors added during the 20 day time window.

### Differences in the Activity of Endogenous BMP Signaling Pathway Between Responsive and Nonresponsive hiPSC Lines

This study data and others indicate that BMP4 is a key driver of pluripotent stem cell differentiation toward non‐neural ectoderm 41, 42. Since one of the hiPSC lines was not responsive to the differentiation method, we investigated whether differences in the endogenous activity of BMP pathway between hiPSC lines could be the underlying mechanism. We first carried out qRT‐PCR analysis which indicated that although the non‐responsive hiPSC line (SB‐Ad3) expressed higher levels of endogenous *BMP4* when compared with the responsive hiPSC line, SB‐Ad2 (Supporting Information Fig. S5), the expression of key receptors (*BMPR1A* and *BMPR1B*) and receptor activated *SMAD1* and *SMAD5* genes that mediate BMP signaling were significantly lower, suggesting that this hiPSC line may be characterized by a much lower level of endogenous BMP activity. This is further corroborated by low expression of two BMP target genes, *ID1* and *JUNB,* which were expressed at a lower level in the nonresponsive hiPSC line when compared with the responsive line. Since both the receptor and effector genes expressions are lower, addition of exogenous BMP4 alone (as in our differentiation methods), is unlikely to activate the pathway in the nonresponsive hiPSC line.

To confirm this further, a BMP reporter plasmid was transfected in both hiPSC lines causing a transient overexpression of BMP specific gene, *ID1* 43. The reporter analyses showed that the SB‐Ad3 iPSC line has a significantly lower endogenous BMP activity compared with SB‐Ad2 iPSC (Supporting Information Fig. S6A). Addition of BMP4 increased the BMP activity of both hiPSCs; however, SB‐Ad3 iPSC still lagged behind SB‐Ad2 (Supporting Information Fig. S6B). Combined addition of BMP4 and SB435142 did not have a significant impact on SB‐Ad2; however, it increased the endogenous BMP activity of Sb‐Ad3 hiPSC to the same levels as SB‐Ad3 (Supporting Information Fig. S6B).

### Specific TGFβ Inhibitor, SB431542, Induces the Differentiation of Nonresponsive hiPSC Lines to Corneal Epithelial Cells

**Figure 6:**
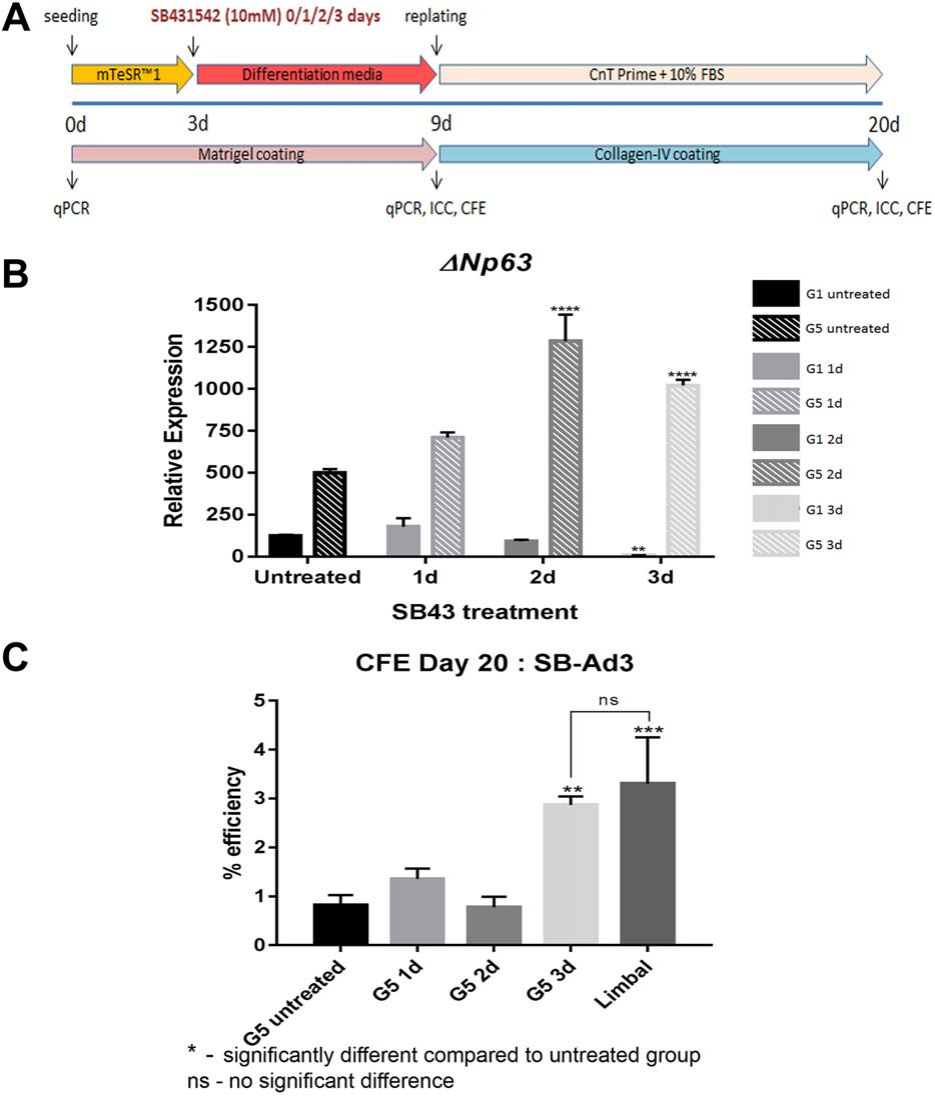
SB431542 exposure during early differentiation enhanced the hiPSC differentiation to corneal epithelial progenitors. Schematic outline for optimization experiment using SB431542 **(A)**. Quantitative real‐time polymerase chain reaction analysis of the putative limbal stem cells (*ΔNp63*) gene for SB‐Ad3 cells from G5 subgroups on day 20 of optimization experiment following different durations of SB431542 exposure **(B)**. Colony forming efficiency assays for the SB431542 exposed and unexposed G5 subgroups on day 20 **(C)**. Data presented as mean ± SEM, *n* = 3. *, statistically different compared with untreated group. **, *p* < .01; ***, *p* < .001; ****, *p* < .0001. ns, no significant difference. Abbreviations: CFE, colony forming efficiency; ICC, immunocytochemistry; FBS, fetal bovine serum; qPCR, quantitative polymerase chain reaction.

Given the low level of BMP receptor and effector expression in the nonresponsive hiPSC line, we aimed to improve the differentiation method by altering the co‐SMAD/r‐SMAD interaction in the cytoplasm. Since co‐SMAD (SMAD4) is shared between TGFβ and BMP pathways 44, 45, we focused on the inhibition of the TGFβ pathway which should lead to an increase in the availability of SMAD4 for the BMP pathway. To achieve this, a selective TGFβ inhibitor, SB431542, which has been reported to drive differentiation away from neuro‐ectoderm 35 and to activate the BMP pathway 46, was used. SB431542 (10 µM) was added for 1, 2, and 3 days to the differentiation media in combination with BMP4, RA, and EGF as detailed in Figure 6A. qRT‐PCR analysis at day 20 indicated the highest expression of *ΔNp63* in groups treated for 2 and 3 days with SB431542 (Fig. 6B). Interestingly, only the group treated for 3 days with SB431542 showed enhanced CFE ability to similar levels observed with human limbal epithelial progenitor cells (Fig. 6C), suggesting that continuous inhibition of TGFβ pathway for 3 days with this specific TGFβ inhibitor, can result in differentiation of nonresponsive hiPSC lines to corneal epithelial progenitor cells (please refer to graphical abstract). It is of interest to note that SB431542 on its own was not able to achieve the upregulation of *ΔNp63* observed after combined addition of BMP4 and SB431542 (Fig. 6B).

## Discussion

Efficient differentiation of a large numbers of hESC and hiPSC for autologous cell replacement therapies using robust and fast protocols has become an important aim for most researchers in the field. In this paper, we report a feeder‐free, two‐step method that results in differentiation of hESC to corneal epithelial progenitors and mature corneal epithelial‐like cells within 20 days. Previous studies in the field have replicated early developmental mechanisms by blocking the TGFβ and Wnt‐signaling pathways with small‐molecule inhibitors and activating fibroblast growth factor (FGF) signaling 27 to generate corneal epithelial‐like progenitor cells capable of terminal differentiation toward mature corneal epithelial‐like cells within 44 days. TGF‐β pathway has been shown to play multiple roles in maintenance of pluripotency and early cell fate decisions. Work done by other groups 47 and this group 48 has shown that low activity of this pathway (either through application of inhibitors or low endogenous activity) results in neuroectodermal default pathway which skews pluripotent stem cells away from non‐neural ectoderm and corneal epithelial differentiation. For this reason, we designed our differentiation protocol to include growth factors and morphogens (BMP4, RA, and EGF) that have been shown to promote non‐neural ectodermal commitment 31, 32, 49, 50 and proliferation of corneal epithelial progenitors. In the second window of differentiation, we attempted to replicate the LSCs niche by coating the cell surfaces with collagen‐IV shown to be the key component of limbal stroma and 51, 52 and feeding the cells with a defined media (CnT‐Prime) which is used to maintain the ex vivo expansion of human corneal epithelial progenitors 53. This two‐step differentiation protocol resulted in successful generation of hESC‐derived corneal epithelial progenitors with colony forming ability similar to limbal epithelial progenitors within a window of 20 days.

Since the main aim of this study was to design robust differentiation protocols for differentiation of hiPSC to corneal epithelial‐like cells for autologous cell replacement therapies, we tested the two‐step differentiation protocol in two hiPSC lines generated and well characterized by our laboratory 54. One of the hiPSC lines was able to generate corneal epithelial progenitors with colony forming ability in response to BMP4, RA, or combined addition of BMP4, RA, and EGF, although at lower levels than hESC. In contrast, the second tested hiPSC line was not able to respond to the step differentiation protocol resulting in low levels of corneal epithelial progenitor generation. Differences in transcriptional and epigenetic profiles between hiPSC lines which are linked to their differentiation capacity are commonly encountered, especially during directed differentiations, where specific molecules were used to alter the pathways of interest. A study published by our group indicated that hiPSC lines that possess higher level of mitochondrial protein CHCHD2, have a less active TGFβ signaling activity, making them more prone to neural differentiation 48. A recent report by Nishizawa et al. also indicated that haematopoietic commitment of hiPSC lines depends on the expression of insulin‐like growth factor 2 (IGF2) 55. Earlier, Fujiwara et al. found variations in the basal cardiomyocyte differentiation efficiency of hiPSC lines which was overcomed by using Cyclosporin‐A 56. Together, these studies suggest that differentiation protocols may need to be adjusted to take into account the endogenous expression of key transcription and growth factors as well as signaling pathways that govern early differentiation steps.

Endogenous BMP signaling activity is different in various hiPSC lines and crosstalk between BMP and TGFβ signaling has also been reported 57, affecting the propensity of each cell line during differentiation process. Given the importance of BMP4 signaling in inhibiting neural differentiation and promoting epidermal commitment of embryonic stem cells 32, 58, 59, we investigated the level of endogenous BMP pathway activity using reporter based assays and qRT‐PCR. These experiments indicated that the nonresponsive hiPSC line had lower level of BMP signaling activity which was caused by a lower expression of effectors and receptors, resulting in low expression of BMP target genes. To restore the differentiation potential we used a specific TGFβ inhibitor, SB431542, which changed the balance of co‐SMADS into the favor of BMP signaling resulting in successful differentiation of the non‐responsive hiPSC to corneal epithelial progenitors. Our findings closely corroborate those published by Shalom‐Feuerstein et al. 42 who reported improved differentiation of hiPSC to epidermal lineages upon addition of SB431542 to BMP4 and ascorbic acid supplemented media. A different small molecule TGFβ inhibitor was used by Mikhailova et al. to guide differentiation of hiPSC to corneal epithelial progenitor cells in combination with a Wnt inhibitor, IWP‐2, and FGF 27. Although SB505124 is reported to be more selective than SB431542 for inhibiting TGFβ signaling 60, the supplementation of the former inhibitor and IWP‐2 alone or in combination with BMP4 in our setting failed to activate expression of key epithelial and LSCs markers, suggesting crosstalk between signaling pathways is essential for guiding differentiation of pluripotent stem cells to corneal epithelial lineages.

Similarly to other published studies in the field, our method generated a high percentage of ΔNp63‐positive cells in the first window of differentiation which went on to further mature to CK3 and CK12 positive corneal epithelial cells. In addition, this study indicated that the hESC and hiPSC derived epithelial progenitors have a high colony forming efficiency which was comparable with limbal epithelial progenitor cells obtained from adult human cornea.

## Conclusion

In summary, this article describes a new two‐step differentiation method with which minor modifications can be applied to generate corneal epithelial progenitor cells in a short time from a large range of hESC and hiPSC. However, further work in animal models of total LSCD needs to be carried out to test the engraftment and functionality of hESC‐ and hiPSC‐derived corneal epithelial progenitor cells.

## Author Contributions

T.A.K.: performed laboratory work, data analysis and interpretation, manuscript writing, final approval of manuscript, S.B., J.C., M.Y., S.A., and H.B.: performed laboratory work and collection and/or assembly of data, final approval of manuscript; A.S., L.A., and F.C.F.: conception/design and fund raising, final approval of manuscript; M.L.: conception/design,data analysis and interpretation,manuscript writing, final approval of manuscript.

## Disclosure of Potential Conflicts of Interest

The authors indicated no potential conflicts of interest.

## Supporting information

Supporting Information Figure 1a

Supporting Information Figure 1B

Supporting Information Figure 1C

Supporting Information Figure 2

Supporting Information Figure 3

Supporting Information Figure 4

Supporting Information Figure 5

Supporting Information Figure 6

Supporting Information Table 1

Supporting Information Table 2
