# Supplementary figures and images for: Differences in the Activity of Endogenous Bone Morphogenetic Protein Signaling Impact on the Ability of Induced Pluripotent Stem Cells to Differentiate to Corneal Epithelial‐Like Cells
Source: Stem Cells. 2017 Dec 21;36(3):337–48. doi: 10.1002/stem.2750 (PMC5839253; doi:10.1002/stem.2750)

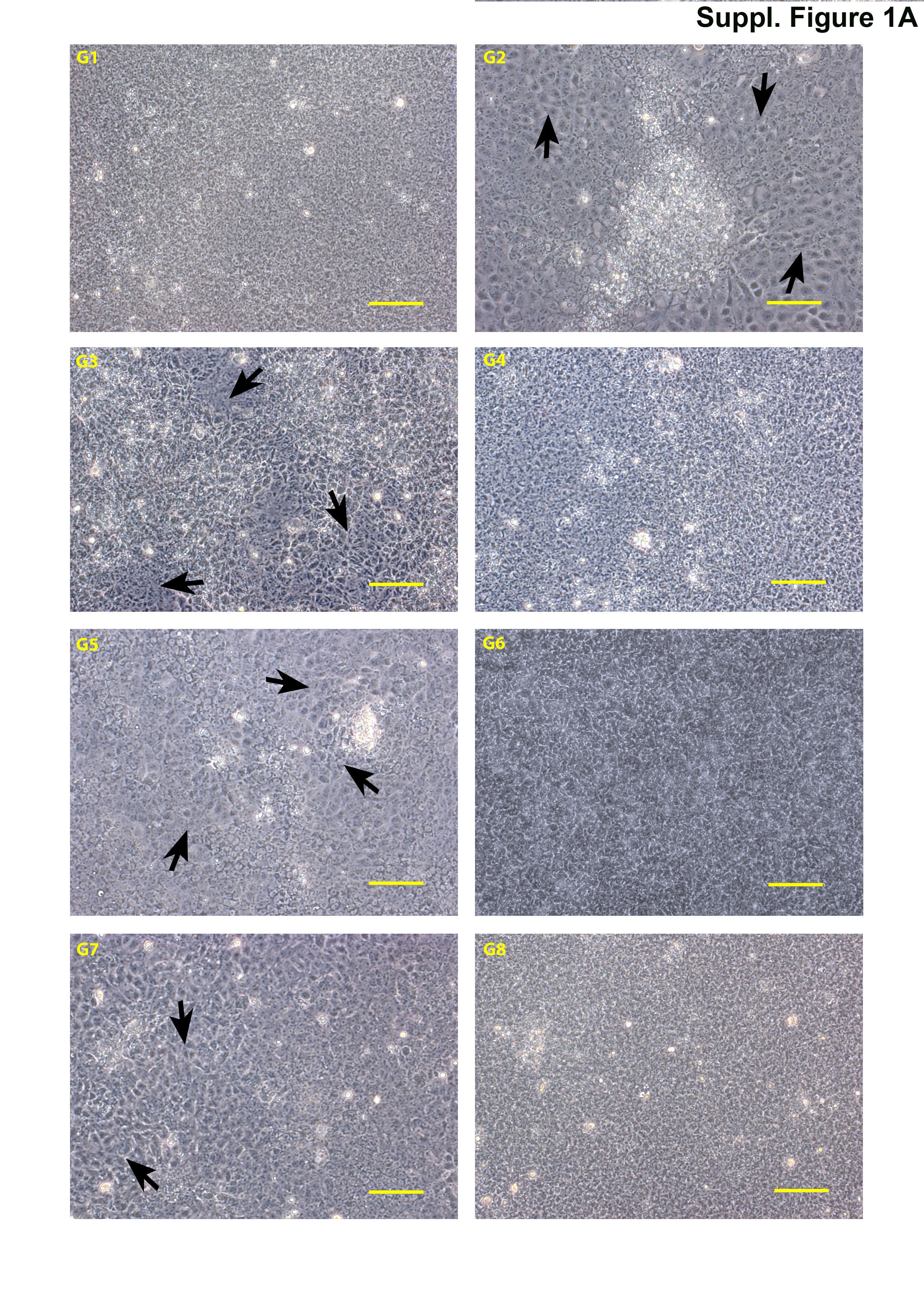

Supplement: Supplementary file 1 — Supporting Information Figure 1a [file STEM-36-337-s001.jpg]

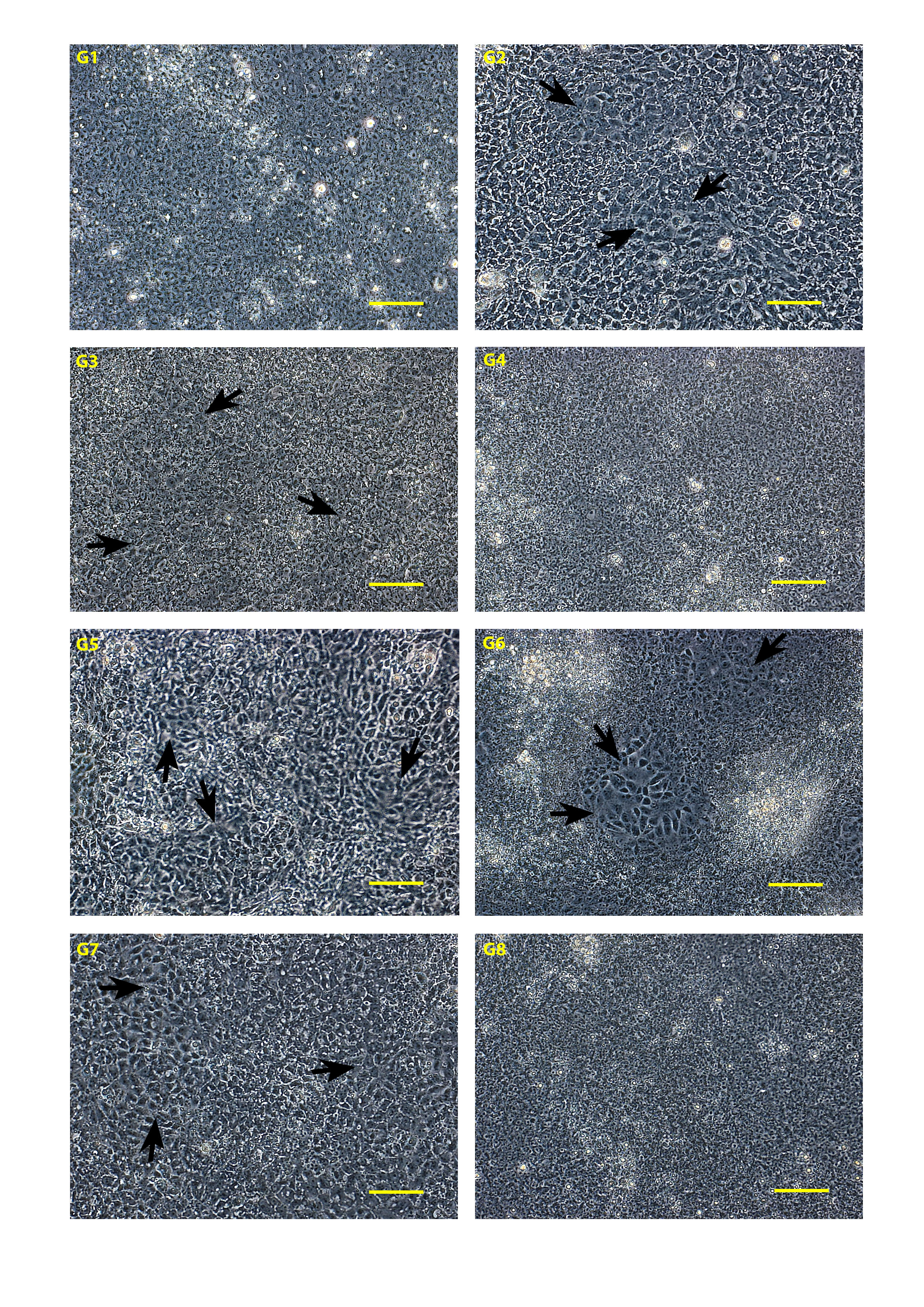

Supplement: Supplementary file 2 — Supporting Information Figure 1B [file STEM-36-337-s002.jpg]

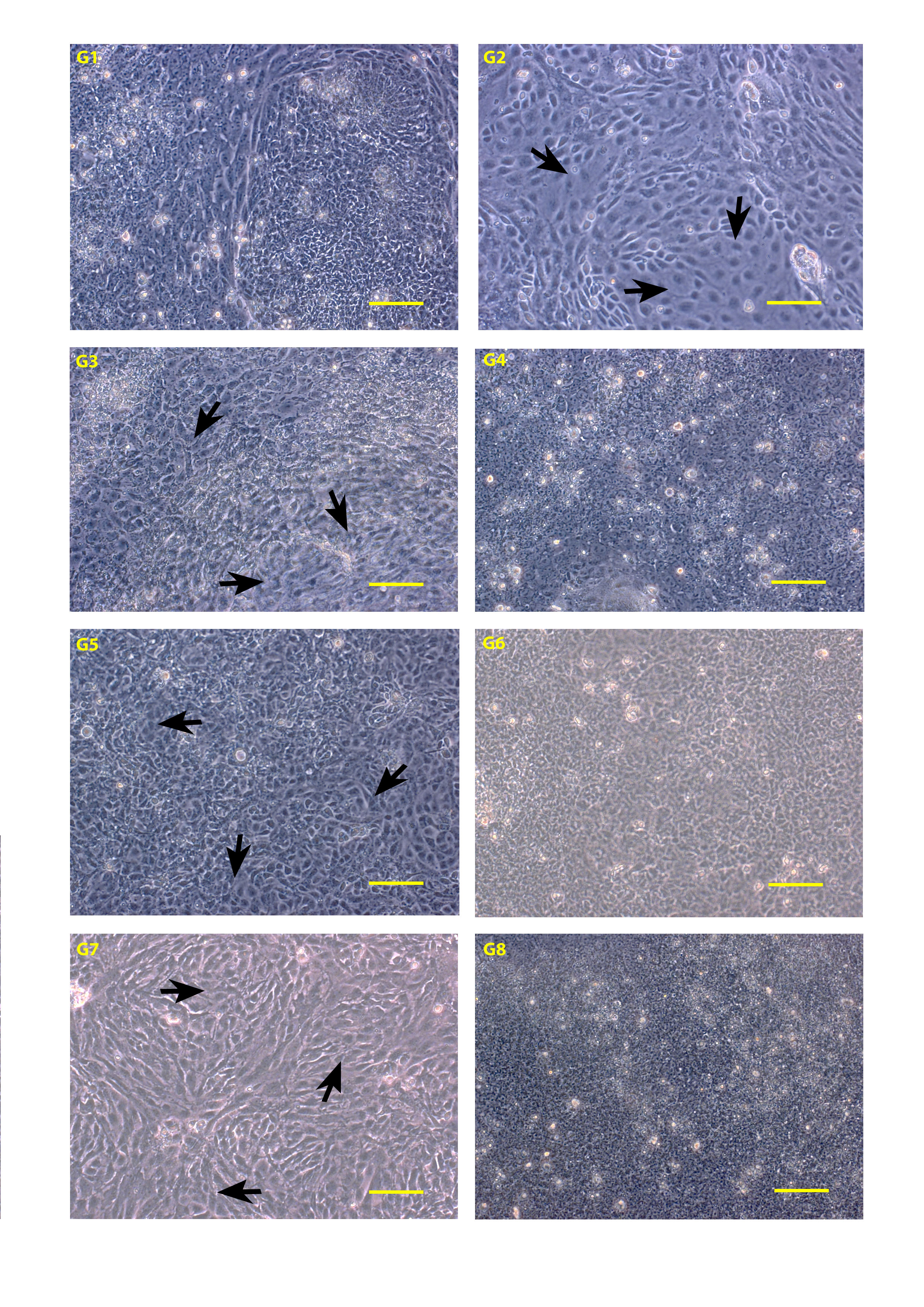

Supplement: Supplementary file 3 — Supporting Information Figure 1C [file STEM-36-337-s003.jpg]

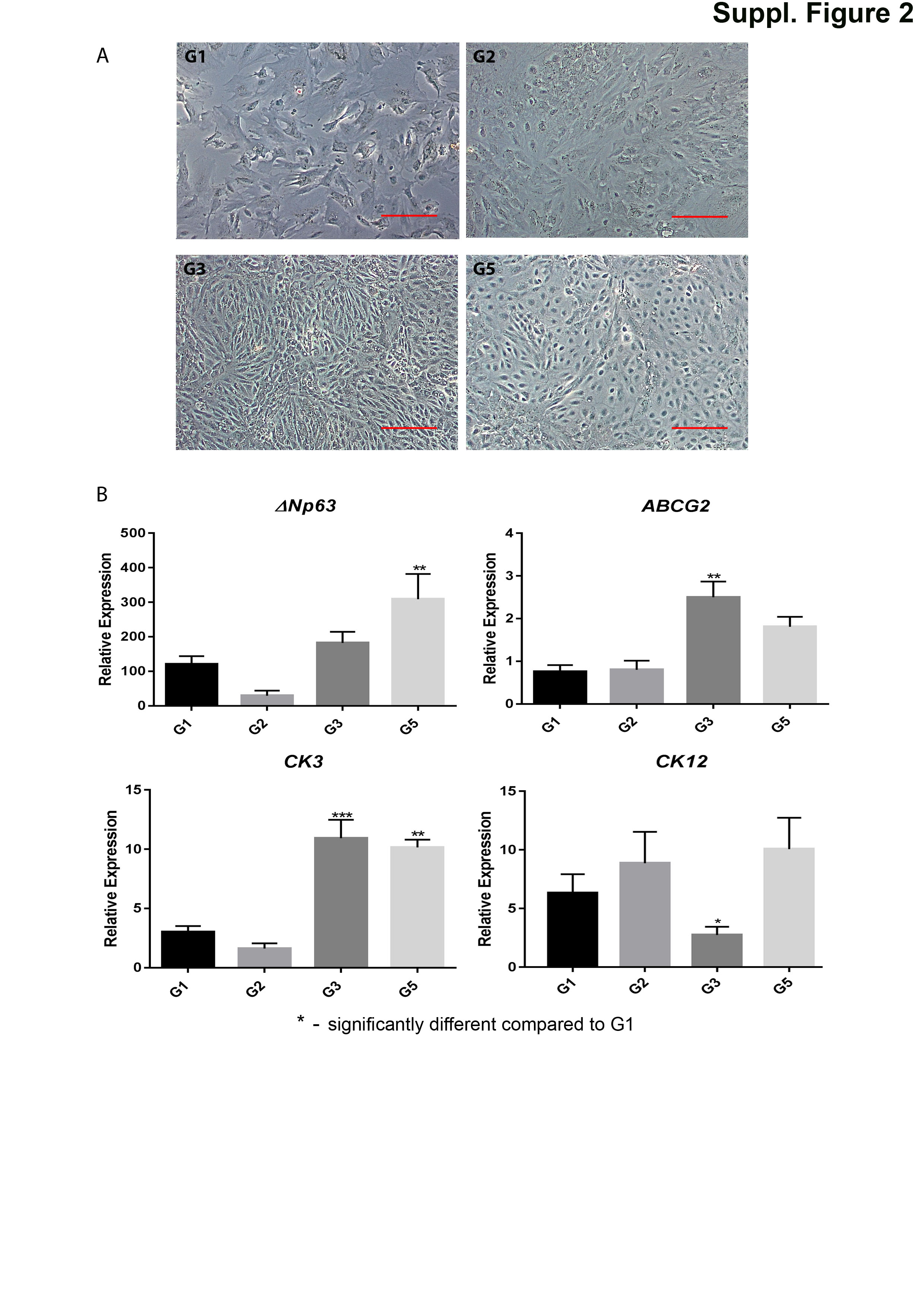

Supplement: Supplementary file 4 — Supporting Information Figure 2 [file STEM-36-337-s004.jpeg]

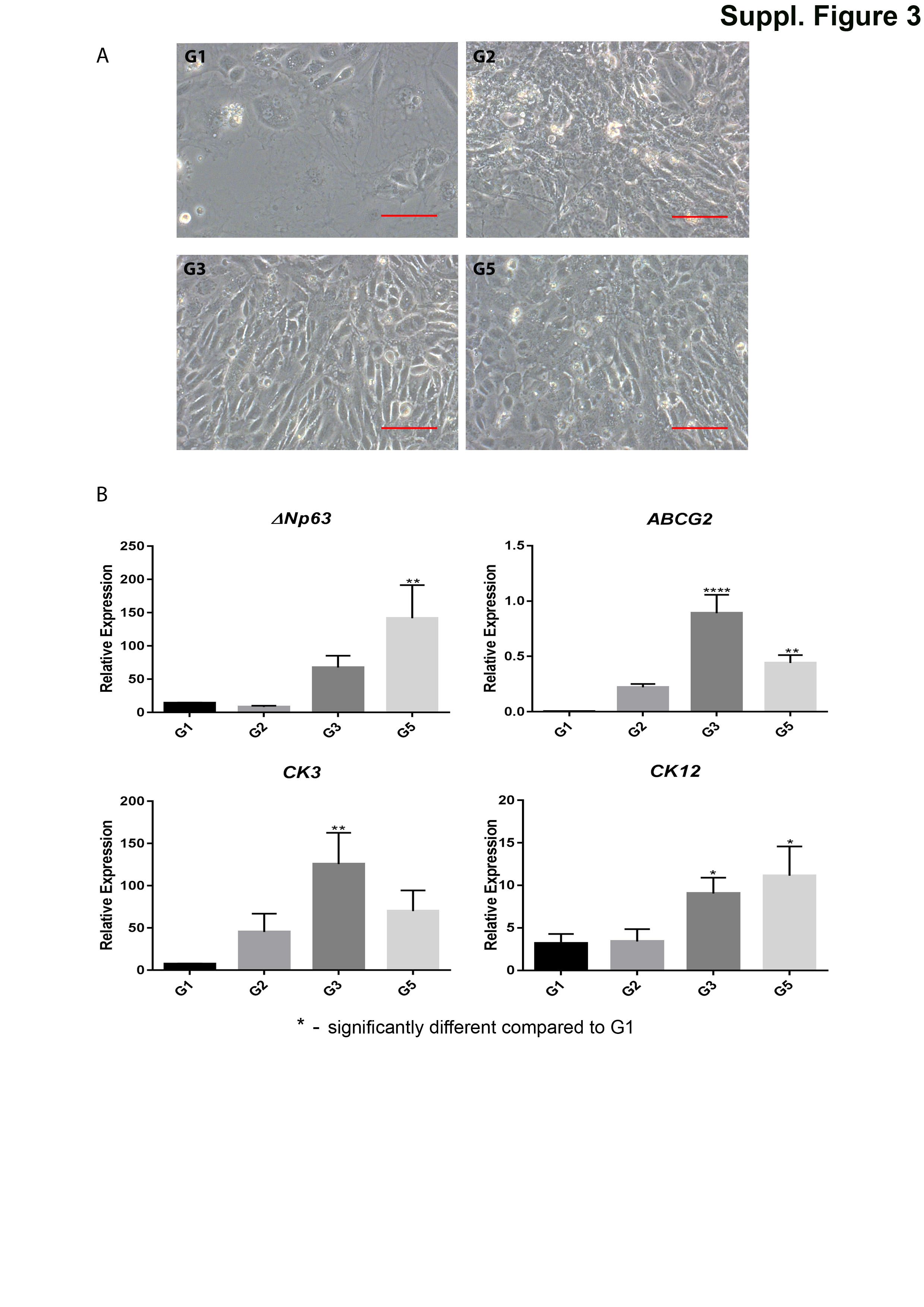

Supplement: Supplementary file 5 — Supporting Information Figure 3 [file STEM-36-337-s005.jpeg]

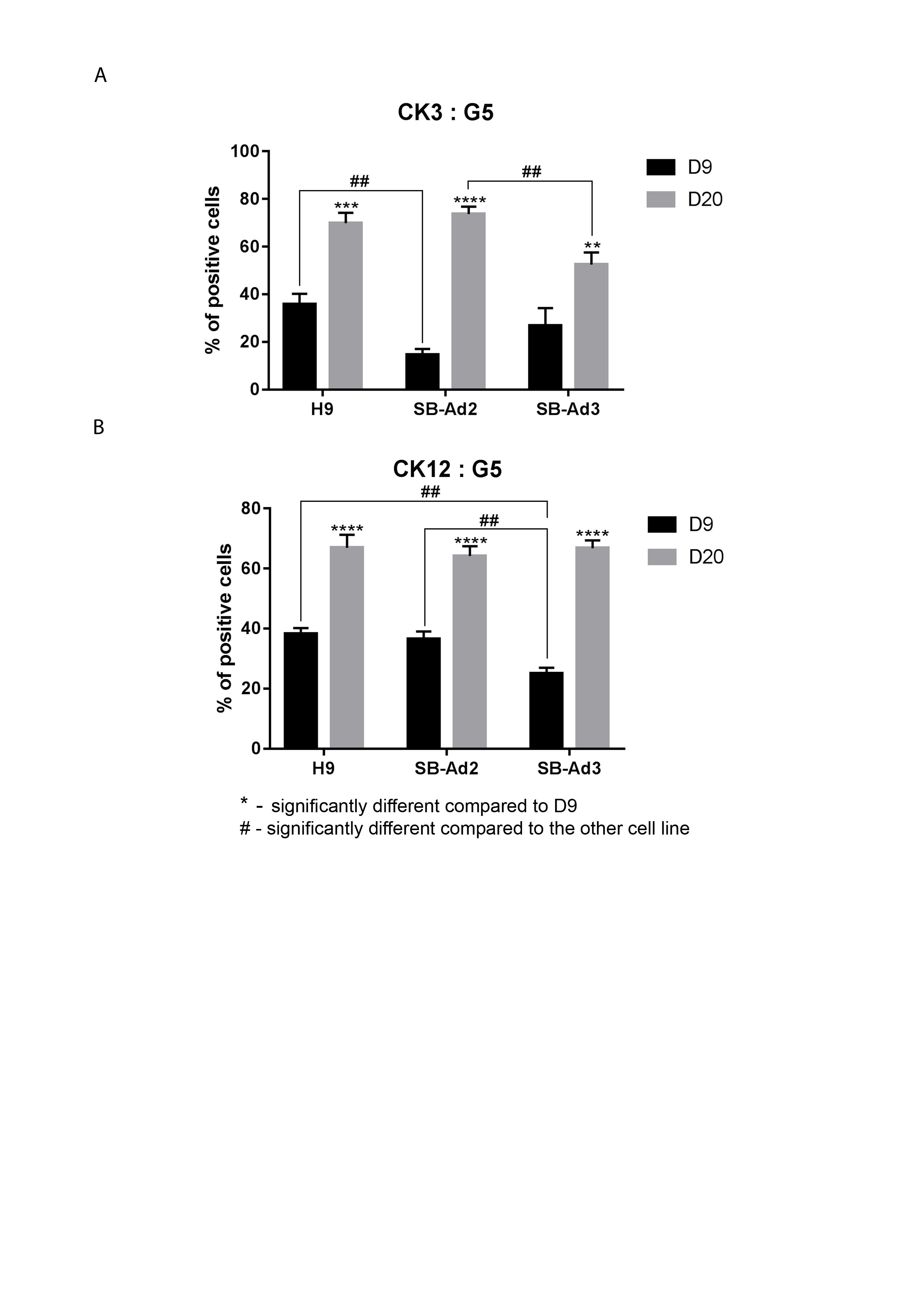

Supplement: Supplementary file 6 — Supporting Information Figure 4 [file STEM-36-337-s006.jpeg]

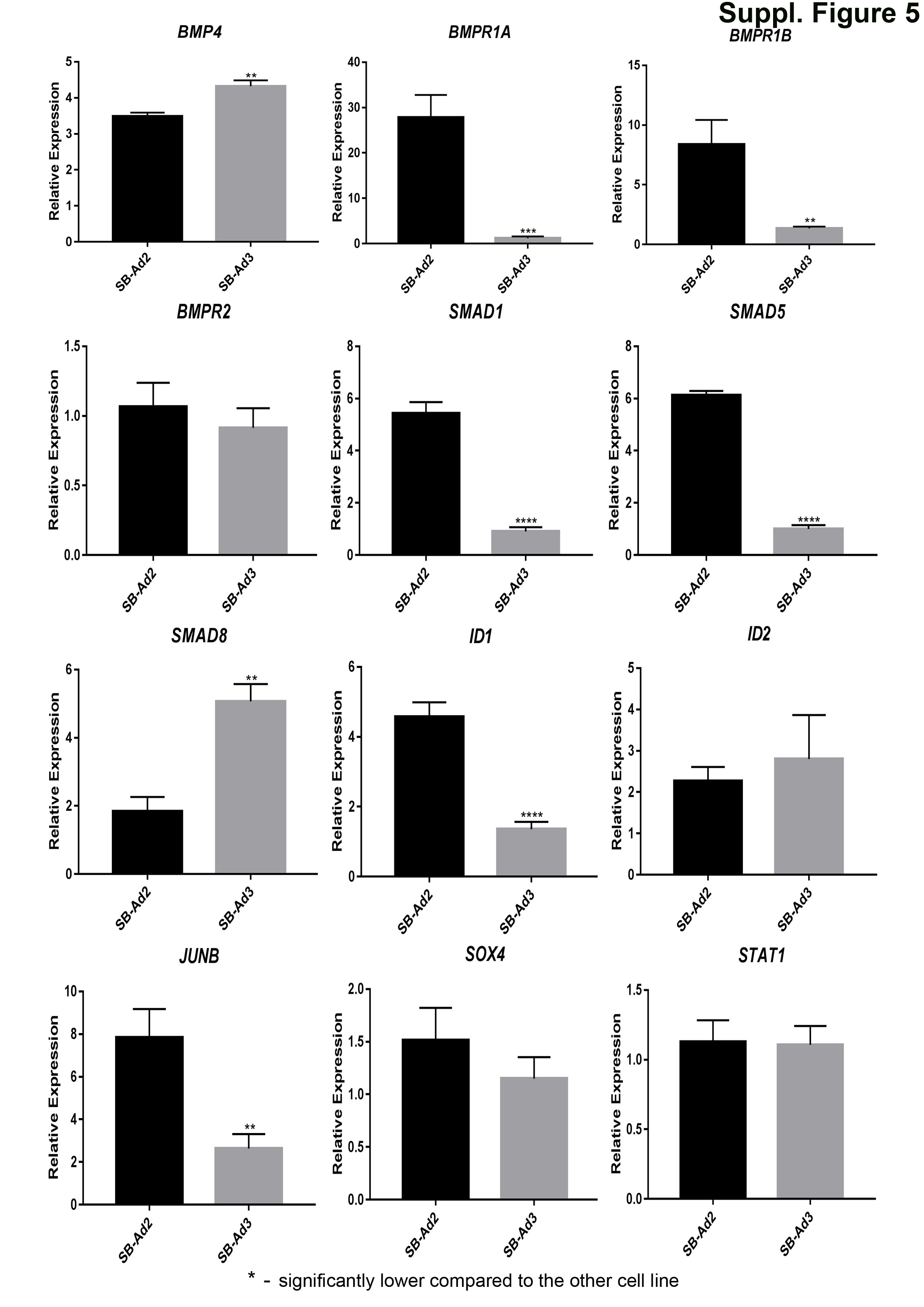

Supplement: Supplementary file 7 — Supporting Information Figure 5 [file STEM-36-337-s007.jpg]

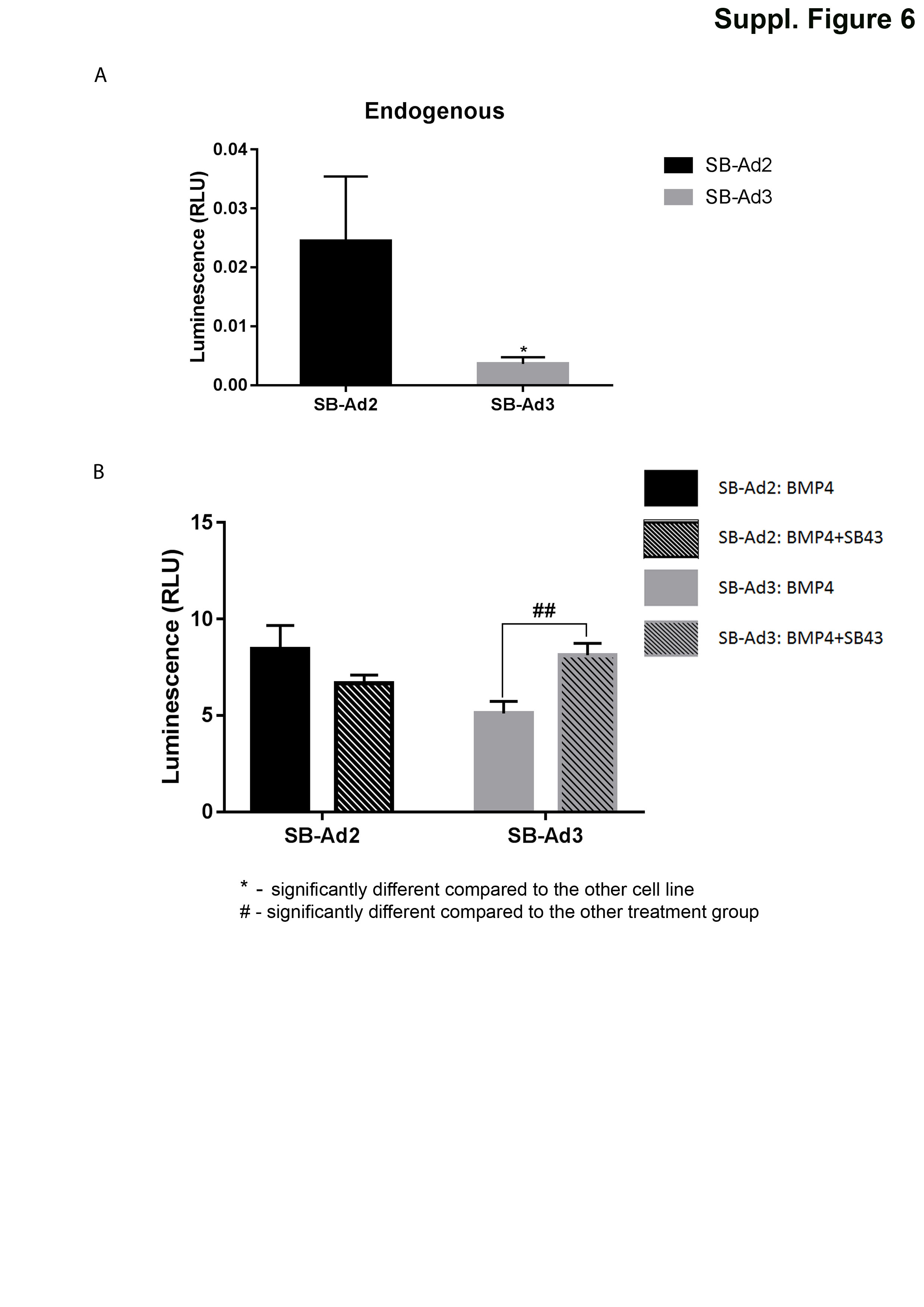

Supplement: Supplementary file 8 — Supporting Information Figure 6 [file STEM-36-337-s008.jpg]
