# Supplementary material for: Differences in the Activity of Endogenous Bone Morphogenetic Protein Signaling Impact on the Ability of Induced Pluripotent Stem Cells to Differentiate to Corneal Epithelial‐Like Cells
Source: Stem Cells. 2017 Dec 21;36(3):337–48. doi: 10.1002/stem.2750 (PMC5839253; doi:10.1002/stem.2750)
Supplement: Supplementary file 9 — Supporting Information Table 1 [file STEM-36-337-s009.docx]

| **Gene Name** | **Primer sequence (5’ – 3’)** |
| --- | --- |
| *ABCG2* | F: CGAGTCTGTTGGTCAATCTC  R: TCCTGTTGCATTGAGTCCTG |
| *BMP4* | F: TCCACAGCACTGGTCTTGAG  R: GGGATGTTCTCCAGATGTTCTT |
| *BRACHYURY* | F: CCCTATGCTCATCGGAACAA  R: CAATTGTCATGGGATTGCAG |
| *CK12* | R: GAAGAAGAACCACGAGGATG  R: TCTGCTCAGCGATGGTTTCA |
| *CK13* | F: GACGCCAAGATGATTGGTTTCCC  R: GACCAGAGGCATTAGAGGTGG |
| *CK3* | F: CGTACAGCTGCTGAGAATGA  R: CTGAGCGATATCCTCATACT |
| *CK8* | F: GATCGCCACCTACAGGAAGCT  R: ACTCATGTTCTGCATCCCAGACT |
| *DeltaNp63* | F: CTGGAAAACAATGCCCAGAC  R: GGGTGATGGAGAGAGAGCAT |
| *E-CADHERIN* | F: CCCGGGACAACGTTTATTAC  R: GCTGGCTCAAGTCAAAGTCC |
| *KRT20* | F: GAGCTGCGAAGTCAGATTAAGG  R: CCTCAGCAGCCAGTTTAGCA |
| *GAPDH* | F: TGCACCACCAACTGCTTAGC  R: GGCATGGACTGTGGTCATGAG |
| *OCT4* | F: TCTCGCCCCCTCCAGGT  R: GCCCCACTCCAACCTGG |
| *PAX6* | F: TCTTTGCTTGGGAAATCCG  R: CTGCCCGTTCAACATCCTTAG |
| *EGF* | F: GCTGTCTGCGTGGTGGTGCT  R: GCTGCCTGGCCATCCTCACC |
| *TGFβ1* | F: GGCCAGATCCTGTCCAAGC  R: GTGGGTTTCCACCATTAGCAC |
| *BMPR1A* | F: TGAAATCAGACTCCGACCAGA  R: TGGCAAAGCAATGTCCATTAGTT |
| *BMPR1B* | F: TCACAAGACGTTTCCTGCGT  R: TGGTGGTGGCATTTACAACG |
| *BMPR2* | F: GGCAGCAGTATACAGATAGGTGA  R: ACTGCCCTGTTACTGCCATT |
| *KLF4* | F: TTACCAAGAGCTCATGCCACC  R: GCGAATTTCCATCCACAGCC |
| *JUNB* | F: ACGACTCATACACAGCTACGG  R: GCTCGGTTTCAGGAGTTTGTAGT |
| *ID1* | F: CTGCTCTACGACATGAACGG  R: GAAGGTCCCTGATGTAGTCGAT |
| *ID2* | F: CCGTGAGGTCCGTTAGGAAA  R: TGAGCTTGGAGTAGCAGTCG |
| *SMAD1* | F: CCGAGCGGCTCAACCC  R: AGTTTGAAGTCCAGAAGAGTAGAA |
| *SMAD4* | F: TGTTGATGACCTTCGTCGCT  R: GGTCTGCAATCGGCATGGTA |
| *SMAD5* | F: CGGCCGAGCTGCTAATAAAG  R: TTCATTGGGTCAAGTCTCGC |
| *SMAD6* | F: CTGAGCCGAGAGAAAGAGCC  R: AAAATGCAGTCCACCGATGC |
| *SMAD7* | F: TTCCTCCGCTGAAACAGGG  R: CCTCCCAGTATGCCACCAC |
| *SMAD 9* | F: CACACAACGCCACCTATCCT  R: ACTGGTCGAAAGTCTGAGTGT |
| *SOX4* | F: GAGTTCCCGGACTACTGCAC  R: GCGCCCTTCAGTAGGTGAAA |
| *STAT1* | F: TTACAAACCTCAAGCCAGCC  R: TGATAGGCAGTAACACGGGG |

List of primers used in qPCR
