# Supplementary material for: Differences in the Activity of Endogenous Bone Morphogenetic Protein Signaling Impact on the Ability of Induced Pluripotent Stem Cells to Differentiate to Corneal Epithelial‐Like Cells
Source: Stem Cells. 2017 Dec 21;36(3):337–48. doi: 10.1002/stem.2750 (PMC5839253; doi:10.1002/stem.2750)
Supplement: Supplementary file 10 — Supporting Information Table 2 [file STEM-36-337-s010.docx]

| **Antibodies** | **React with** | **Developed in** | **Dilution** | **Cat. No. / Company** |
| --- | --- | --- | --- | --- |
| CK3 primary | Rabbit, cow, human | Mouse | 1:100 | [AE5] Ab77869 / Abcam |
| P40 (p63 delta) primary | Human, mouse, rat, bovine | Rabbit | 1:100 | NBP2-29467 / Novusbio |
| CK12 primary | human | Rabbit | 1:100 | NBP2-34843/ Novusbio |
| CK13 primary (AF 647 conjugated) | Human, mouse | Rabbit | 1:200 | [EPR3671] Ab198585 / Abcam |
| Pax6 primary | Mouse, rat, sheep, cow, dog, human, rhesus monkey | Rabbit | 1:50 | Ab5790 / Abcam |
| ANTI-MOUSE IgG (WHOLE MOLECULE) FITC CONJUGATE | Mouse | Goat | 1:800 | F2012 / Sigma |
| Anti-Rabbit IgG (whole molecule)-FITC | Rabbit | Goat | 1:800 | F9887 / Sigma |
